# Cell-free Nucleic Acid as Promising Diagnostic Biomarkers for Gastric Cancer: a Systematic Review

**DOI:** 10.7150/jca.92704

**Published:** 2024-03-25

**Authors:** Qun Zhang, Zhouyuan Du, Xiaoxiao Wang, Fen Li, Yi Liu, Jingjing Sun, Lin Zhang, Yong Xiao, Xiaoming Lu, Haixin Yu, Tao Liu

**Affiliations:** Department of Digestive Surgical Oncology, Union Hospital, Tongji Medical College, Huazhong University of Science and Technology, Wuhan 430022, China.

**Keywords:** gastric cancer, early detection, cfRNA, cfDNA, biomarker

## Abstract

**Background:** Gastric cancer (GC) is a common malignancy with early detection being crucial for survival. Liquid biopsy analysis using cell-free nucleic acid is a preferred method for detection. Hence, we conducted a systematic review to assess the diagnostic efficacy of cell-free nucleic acid markers for GC.

**Methods:** We searched PubMed and ISI Web of Science databases for articles that conformed to our inclusion and exclusion criteria from 2012 to 2022. The following information was abstracted: first author, year of publication, country/region, age, male proportion, tumor stage for cases, specimen type, measurement method, targeted markers and diagnostic related indicators (including sensitivity, specificity, AUC, P-value).

**Results:** Fifty-eight studies examined cell-free RNAs (cfRNAs) with a total of 62 individual circulating markers and 7 panels in serum or plasma, while 21 studies evaluated cell-free DNAs (cfDNAs) with 29 individual circulating markers and 7 panels. For individual cfRNAs, the median (range) sensitivity and specificity were 80% (21% - 98%) and 80% (54% - 99%), respectively. The median (range) sensitivity and specificity for cfRNA panels were 86% (83% - 90%) and 75% (60% - 98%), respectively. In comparison, the median (range) sensitivity and specificity reported for individual cfDNAs were 50% (18% - 96%) and 93% (57% - 100%), respectively, while cfDNA panels had a median (range) sensitivity and specificity of 85% (41% - 92%) and 73.5% (38% - 90%), respectively. The meta results indicate that cfRNA markers exhibit high sensitivity (80%) and low specificity (80%) for detecting GC, while cfDNA markers have lower sensitivity (59%) but higher specificity (92%).

**Conclusions:** This review has demonstrated that cell-free nucleic acids have the potential to serve as useful diagnostic markers for GC. Given that both cfRNA and cfDNA markers have shown promising diagnostic performance for GC, the combination of the two may potentially enhance diagnostic efficiency.

## 1. Introduction

Gastric cancer (GC) is one of the most widespread malignant tumors globally, responsible for over a million new cases and an estimated 769,000 deaths in 2020 1. It ranks fifth for incidence and fourth for mortality worldwide, with Asia and Eastern Europe experiencing the highest burden of the disease, particularly in Japan and China 2. Prognosis worsens as the tumor advances, with metastatic gastric cancer patients having a five-year survival rate of only 3.1% compared to a five-year survival rate of over 90% in patients with early gastric cancer 3. Thus, early diagnosis and prompt treatment are crucial in improving the survival and prognosis of GC.

The notion of liquid biopsy was first introduced for circulating tumor cells (CTC) a decade ago and was subsequently extended to include circulating tumor DNA (ctDNA) and circulating cell-free RNA (cfRNA) 4. It is widely acknowledged that the bulk of such ctDNA stems from apoptotic and necrotic tumor cells that discharge their fragmented DNA into circulation. Essentially, ctDNA bears the genetic flaws that are identical to those of the tumor cells they are derived from 5. In addition to mutation analysis, trustworthy tests have been developed over the last few years for the assessment of epigenetic alterations, including DNA methylation 4. Compared to ctDNA, cfRNA is unstable and has a short half-life, but its broad classification and distribution in peripheral blood and other bodily fluids make it a superior biomarker for liquid biopsy 6. MicroRNAs (miRNAs) are typically the primary targets of cfRNA. Additionally, long noncoding RNA (lncRNA), circular RNA (circRNA), and transfer RNA (tRNA)-derived fragments (tRF) are also novel biomarkers for cancer diagnosis 7-9.

Currently, cell-free nucleic acid is widely acknowledged as a superior biomarker class for the liquid biopsy analysis of GC. The aim of this systematic review is to provide a comprehensive summary of published articles on the utility of cell-free nucleic acid as a biomarker for GC detection, with a specific emphasis on its potential for early disease detection, which is an indispensable factor in enhancing patient prognosis and management.

## 2. Methods

We conducted this systematic review following a predefined protocol and adhering to the reporting standards set out in the PRISMA statement 10. Seventy-nine studies were included in the meta-analysis. Summary receiver operator characteristics (SROC) for cf-RNAs and cf-DNAs were drawn. Calculations were made by using bivariate mixed-effects model developed by von Houwelingen for treatment trial meta-analysis and then modified for synthesis of diagnostic test data, which is in order midas in STATA 17.0. As we solely used data from previously published literature, no ethical approval or patient informed consent was necessary.

### 2.1 Search strategy

We searched PubMed and ISI Web of Science databases for articles that conformed to our inclusion and exclusion criteria from 2012 to 2022. The combination keywords we used to separately search for DNA and RNA markers were as follows: [(gastric OR stomach) AND (cancer OR carcinoma OR neoplasm OR tumor OR adenocarcinoma OR squamous carcinoma OR malignancy) AND (cell-free RNA OR cfRNA OR mRNA fragment OR microRNA* OR miRNA* OR long noncoding RNA OR lncRNA OR circular RNA OR circRNA OR transfer RNA OR tRNA noncoding RNA OR ncRNA OR RNA) AND (detection OR diagnosis OR biomarker OR marker OR sensitivity OR specificity OR area under the curve OR AUC) AND (blood OR serum OR plasma)] for cf-RNA markers, and [(gastric OR stomach) AND (cancer OR carcinoma OR neoplasm OR tumor OR adenocarcinoma OR squamous carcinoma OR malignancy) AND (Circulating Tumor DNA OR ctDNA OR cell-free DNA OR cfDNA OR DNA) AND (detection OR diagnosis OR biomarker OR marker OR sensitivity OR specificity OR area under the curve OR AUC) AND (blood OR serum OR plasma)] for cf-DNA markers.

### 2.2 Eligibility criteria

In this systematic review, eligible studies were selected based on the following inclusion criteria: (1) reported at least one of the diagnostic values of cfDNA or cfRNA detection in GC patients or able to calculate it from published data; (2) samples collected from peripheral blood; (3) clearly stated the techniques and target gene used in the study; (4) studies must include negative controls. In addition, the following exclusion criteria were also applied: (1) studies published in languages other than English; (2) repeated or overlapping publications that included the same population and gene; (3) studies with a poor sample size (≤20); (4) studies that were letters, editorials, case reports or case series; (5) non-human studies; (6) experiments only based on cell lines rather than clinical samples; (7) studies using treated cases before sampling or disease controls were also excluded.

### 2.3 Data extraction and statistical analysis

Two investigators (QZ and ZD) independently performed data extraction of all included studies. The following information was abstracted: first author, year of publication, country/region, age, male proportion, tumor stage for cases, specimen type, measurement method, targeted markers and diagnostic indicators (including sensitivity, specificity, AUC, P-value). Consensus was obtained by discussion in case of any disagreement. The analytical software STATA 17.0 was used to analyze the diagnostic value. Sensitivity, specificity, area under curve (AUC) were calculated.

### 2.4 Quality assessment

The two authors independently assessed the risk of bias and applicability concerns of the included studies using Quality Assessment of Diagnostic Accuracy Studies-2 (QUADAS-2) 11. Any disagreement was settled by further discussion among the authors.

## 3. Results

### 3.1 Literature search result

**Figure 1:**
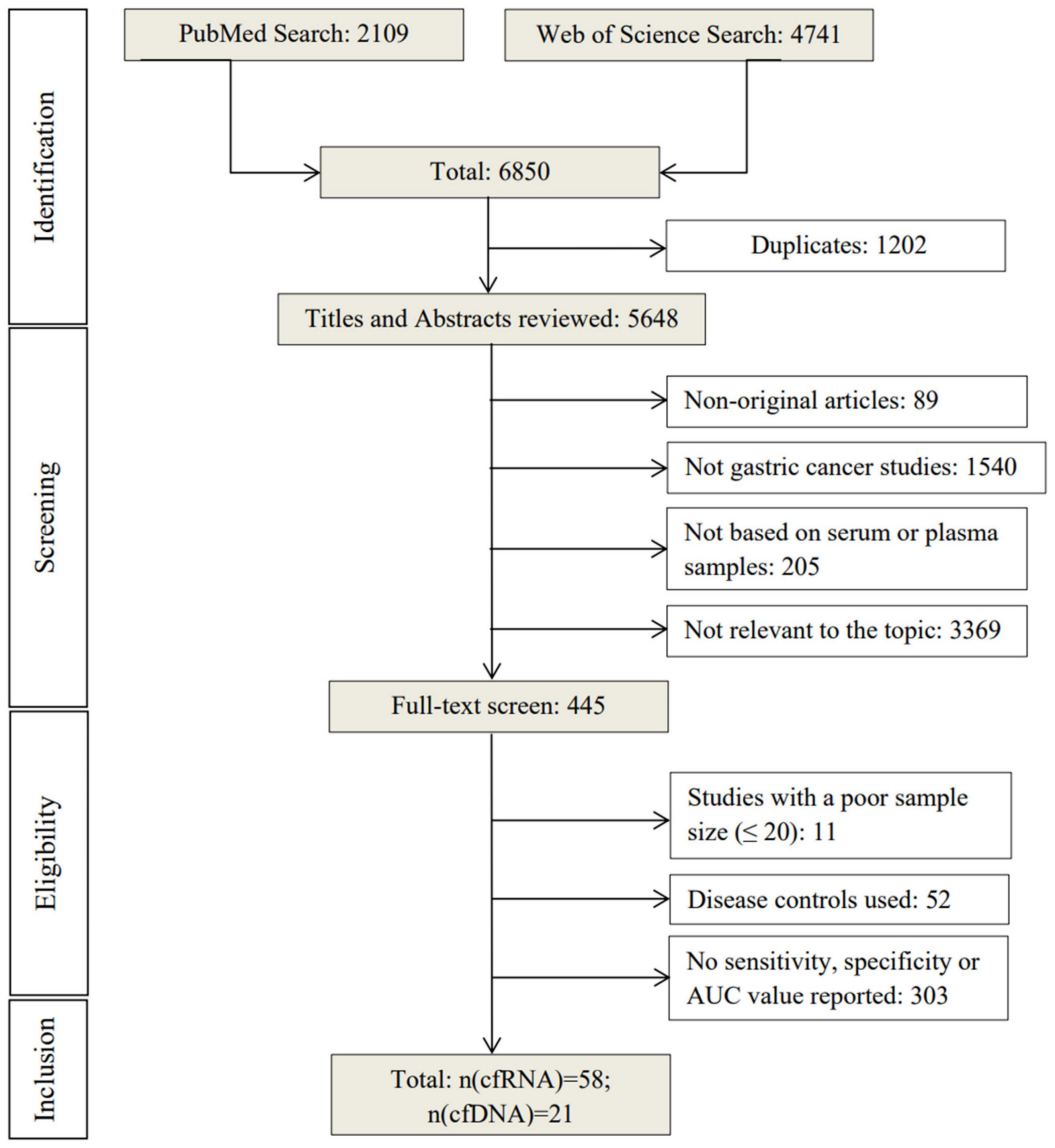
Overview of the literature search process (up to 17th of November 2022).

Initial electronic search retrieved 6850 articles from PubMed (2109) and Web of Science (4741) using the search terms mentioned above (Figure 1). After removing duplicates (n=1202), the remaining 5648 articles were screened by title and abstract based on the exclusion criteria. 5203 articles were excluded and 445 were selected for full-text reading. Among these, 366 were excluded for using disease controls or not reporting sensitivity, specificity, or AUC values, leaving a total of 79 studies 12-59 for evaluating the diagnostic performance of cell-free nucleic acid for GC 60-90.

### 3.2 Study quality and characteristics

QUADAS-2 was carried out for the 79 included studies for quality assessment (Figures S1, S2, S3 and S4). Study quality assessment was completed by two reviewers (QZ and HY) independently. Any initial inconsistencies were resolved by further discussion between the investigators. No risk of bias or applicability concern was found in the reference standard domain, and the flow and timing domain.

The two reviewers (QZ and ZD) independently carried out quality assessment of the 79 included studies (see Supplementary Figures S1, S2, S3 and S4). Any inconsistencies were resolved through further discussion. No bias or applicability concerns were found in the reference standard domain, as well as in the flow and timing domain.

All 79 included studies were case-control studies that collected blood samples after disease diagnosis. Of these, 58 studies evaluated cfRNAs and 21 studies evaluated cfDNAs. Within the cfRNA studies, 55 articles evaluated individual cfRNAs, including 34 miRNAs, 10 lncRNAs, eight circRNAs, and four tRFs (Table 1). Seven studies assessed RNA panels, five of which were miRNA panels (Table 2). Among the cfDNA studies, we reviewed 20 articles on individual cfDNAs, including three on DNA hypermethylation (Table 3). Seven studies evaluated cfDNA panels, three of which were hypermethylated panels (Table 4). Information on each study, such as the number of cases and controls, mean or median age, male proportion, specimen type, tumor stage, and diagnostic indicators, was summarized in Tables 1, 2, 3, and 4. Tables 1 and 3 also presented the p-value for testing the difference of each individual RNA between cases and controls or the statistical significance of AUC values.

Twenty-five studies analyzed plasma samples and 33 studies analyzed serum samples for cfRNA. Overall, 58 studies evaluated 62 individual circulating cfRNA markers and seven cfRNA panels in serum or plasma. All enrolled cfRNA studies used quantitative real time polymerase chain reaction (qRT-PCR) to detect cfRNAs concentrations. The normalization methods for the expression of RNAs were not uniform. For example, miR-39, U6 snRNA, miR-16, 18S rRNA, GAPDH, β-actin, and snord47 were being used as reference standards for data normalization (Table S1). Thirteen studies analyzed plasma samples and eight studies analyzed serum samples for cfDNA. The cfDNAs were isolated by different extraction kits among the included studies (Table S2). Twenty-one studies reported 29 individual circulating cfDNA markers and seven cfDNA panels in serum or plasma. Most of 21 studies quantified methylation levels using Methylation Specific PCR (MSP). Only one study conducted by Hideura E et al 76. additionally used digital PCR to quantify methylation level.

### 3.3 Diagnostic efficiency of cfRNAs and cfDNAs

**Figure 2:**
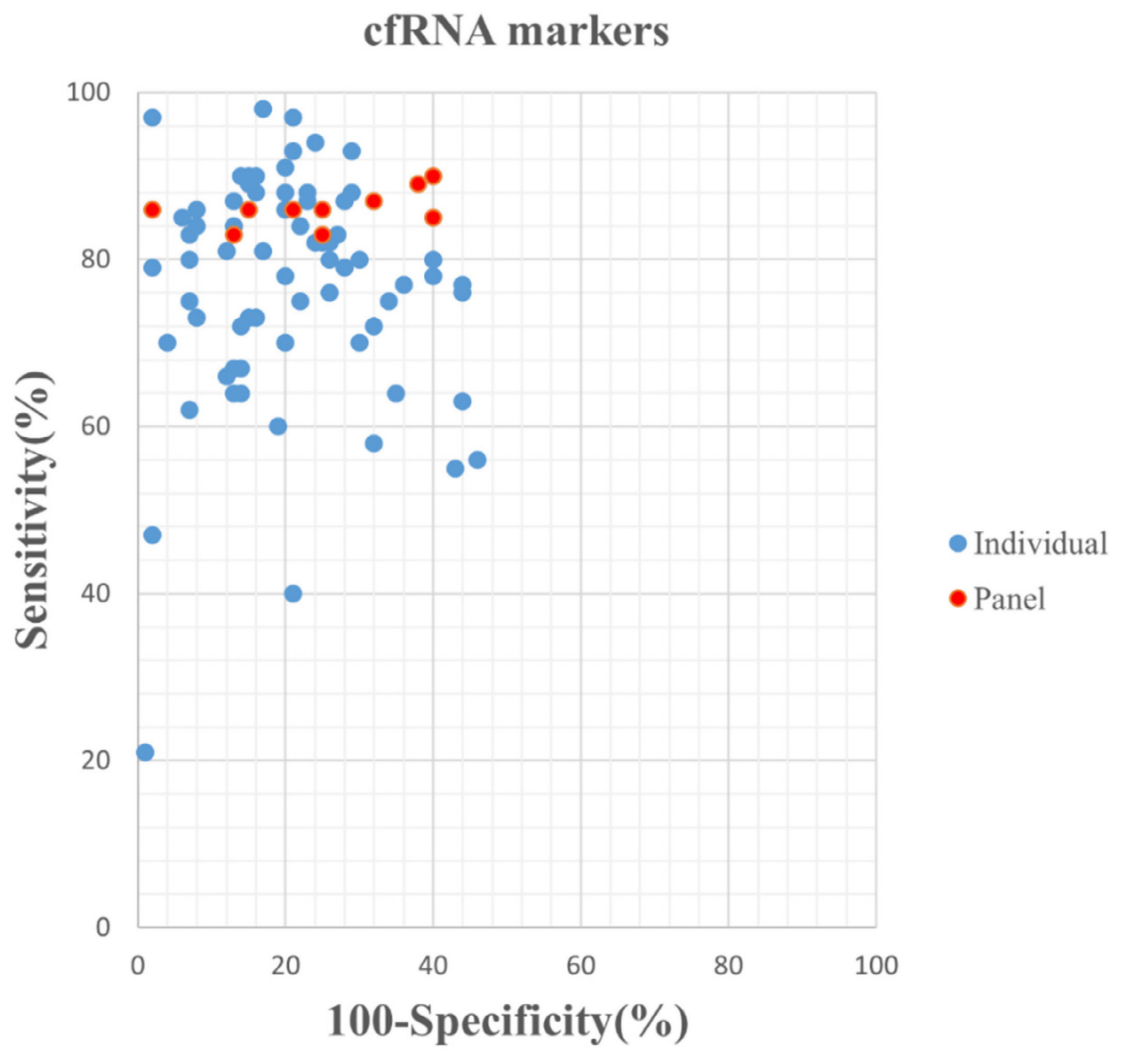
Graphical representation of sensitivity vs specificity of analyzed cfRNA markers. Sensitivity is plotted on the y-axis while on the x-axis the false-positive rate is presented (100-Specificity).

The 58 included studies reported a total number of 84 cfRNAs with the diagnostic potential for GC, of which, seven miRNAs were reported in more than two studies (Table S3). The panels ranged from two to 12 miRNAs, with the smallest and largest panel sizes being two and 12 miRNAs, respectively. Figure 2 presents an overview of the diagnostic performance of all reported cfRNAs and cfRNA panels. For individual cfRNAs, the median (range) reported sensitivity and specificity were 80% (21%-98%) and 80% (54%-99%), respectively.

**Figure 3:**
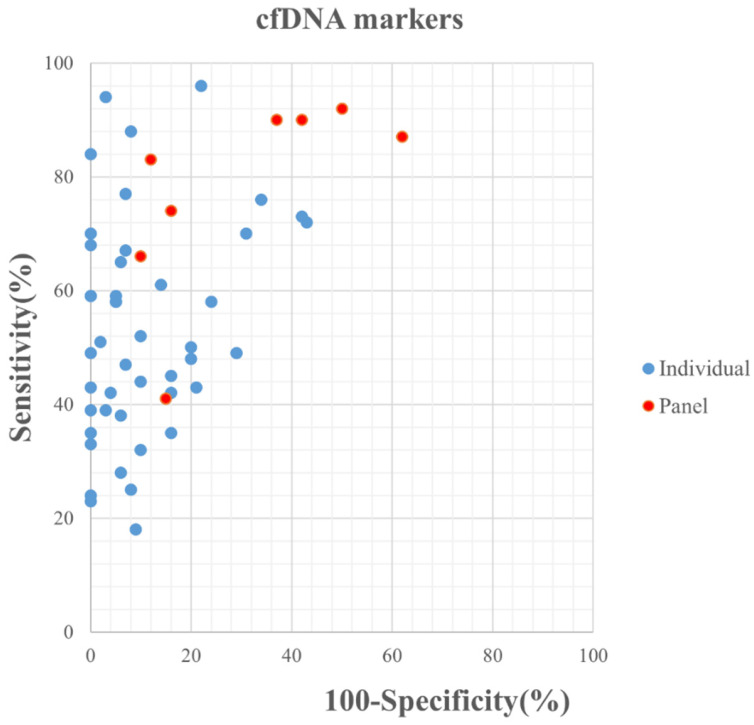
Graphical representation of sensitivity vs specificity of analyzed cfDNA markers. Sensitivity is plotted on the y-axis while on the x-axis the false-positive rate is presented (100-Specificity).

The median (range) reported sensitivity and specificity of RNA panels were 86% (83%-90%) and 75% (60%-98%), respectively. The 21 included studies reported a total number of 32 cfDNAs with the diagnostic potential for GC, of which, seven cfDNAs were reported in more than two studies (Table S4). Five reported cfDNA panels for GC diagnosis contained three number of cfDNAs and two panels contained two individual cfDNAs. An overview of the diagnostic performance of all reported cfDNAs and cfDNA panels was shown in Figure 3. The reported sensitivity and specificity for individual cfDNAs were 50% (range: 18%-96%) and 93% (range: 57%-100%), respectively. The median (range) reported sensitivity and specificity of cfDNA panels were 85% (41%-92%) and 73.5% (38%-90%), respectively. Overall, the sensitivity of cfRNA and cfDNA panels appeared to be better than that of individual cfRNAs or cfDNAs, but the specificity was lower.

Among the included studies of cfRNAs, two enrolled patients with stages I-III 12,39, three enrolled patients with earlystage disease, specifically stages I-II 35,37,48, and the remaining studies included patients with stages I-IV. Subgroup analyses were conducted in four studies 34,35,45,46. Li et al. 35 evaluated the diagnostic efficiency of miR-199a-3p for stages I and II GC, reporting a sensitivity and specificity of 76% and 74%, respectively. They also reported the diagnostic efficiency of miR-199a-3p for stages I-IV GC, with a sensitivity of 80% and specificity of 74% 34. Roy et al. 45 investigated an eight-circRNA panel for early GC, reporting a sensitivity of 90%, specificity of 60%, and AUC of 0.82. This panel was equally effective in diagnosing both early GC and stage I-IV GC. Saliminejad et al. 46 conducted a nested case-control study exploring the diagnostic efficacy of a three-miRNA panel in plasma for GC occurring at stages I-II, III-IV, and I-IV, reporting AUC values of 0.83, 0.93, and 0.92, respectively.

Of the included studies of cfDNAs, only two enrolled early-stage patients 73,76, and only one performed stage-specific analysis 84. Saliminejad et al. 84 evaluated the diagnostic efficiency of four cfDNAs (P16, RASSF1A, RPRM, RUNX3) for GC and found no significant difference in AUC values among stages I-II, III-IV, and I-IV (Table 3).

Seven miRNAs were reported in at least two studies, with miRNA-21 being the most commonly reported in five studies, followed by miR-421, miR-222, miR-106a, miR-25, miR-93, and miR-199a-3p, all of which were reported in two studies (Table S3). SEPT9 was the most frequently reported cfDNA (Table S4), with sensitivity ranging from 18% to 52% (median sensitivity 41.5%), specificity ranging from 85% to 96% (median specificity 90.5%), and AUC values ranging from 0.65 to 0.77 (median AUC value = 0.7).

### 3.4 Overall comparison of cfRNA and cfDNA markers for GC detection

**Figure 4:**
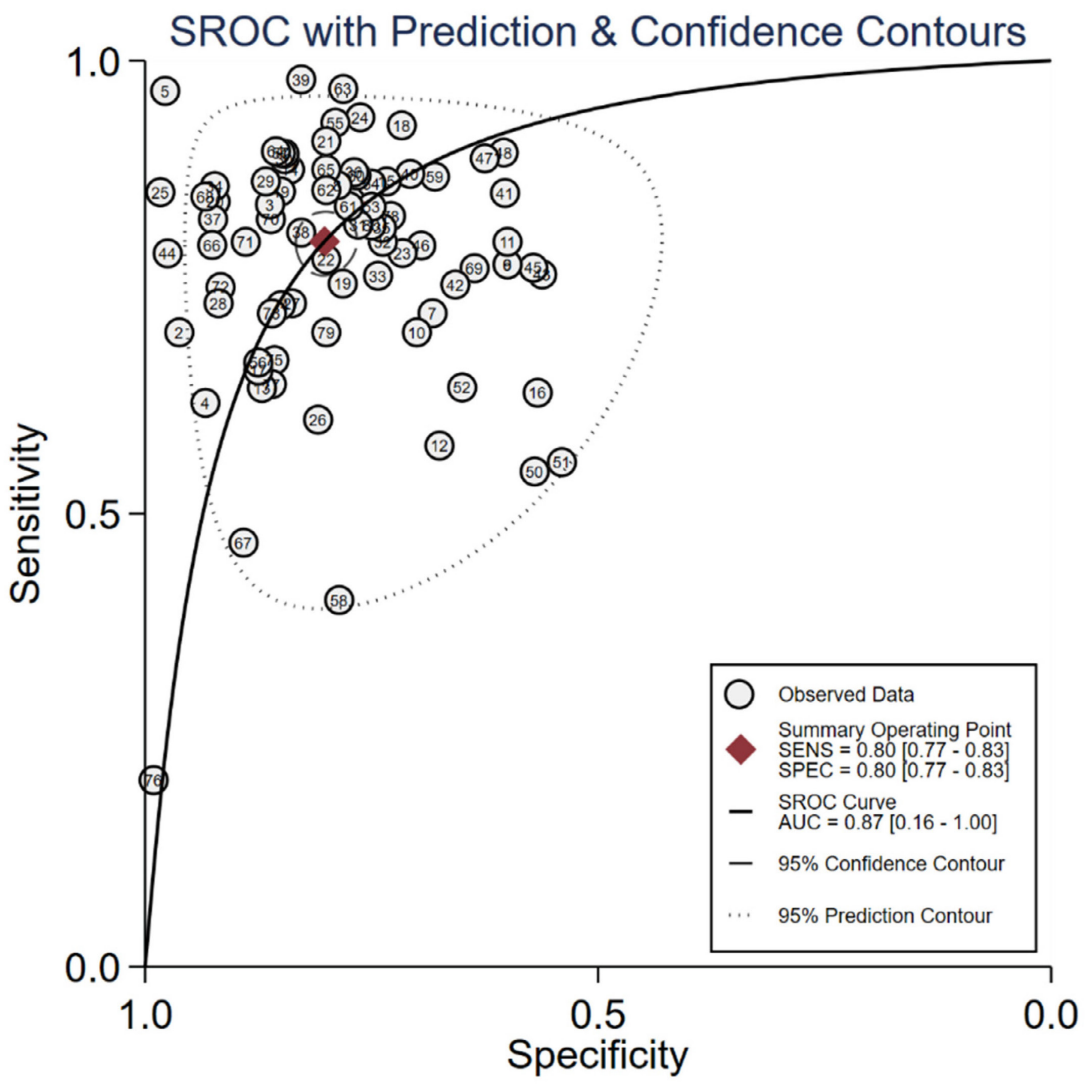
Summary of AUC of cfRNA markers for the diagnosis of gastric cancer.

**Figure 5:**
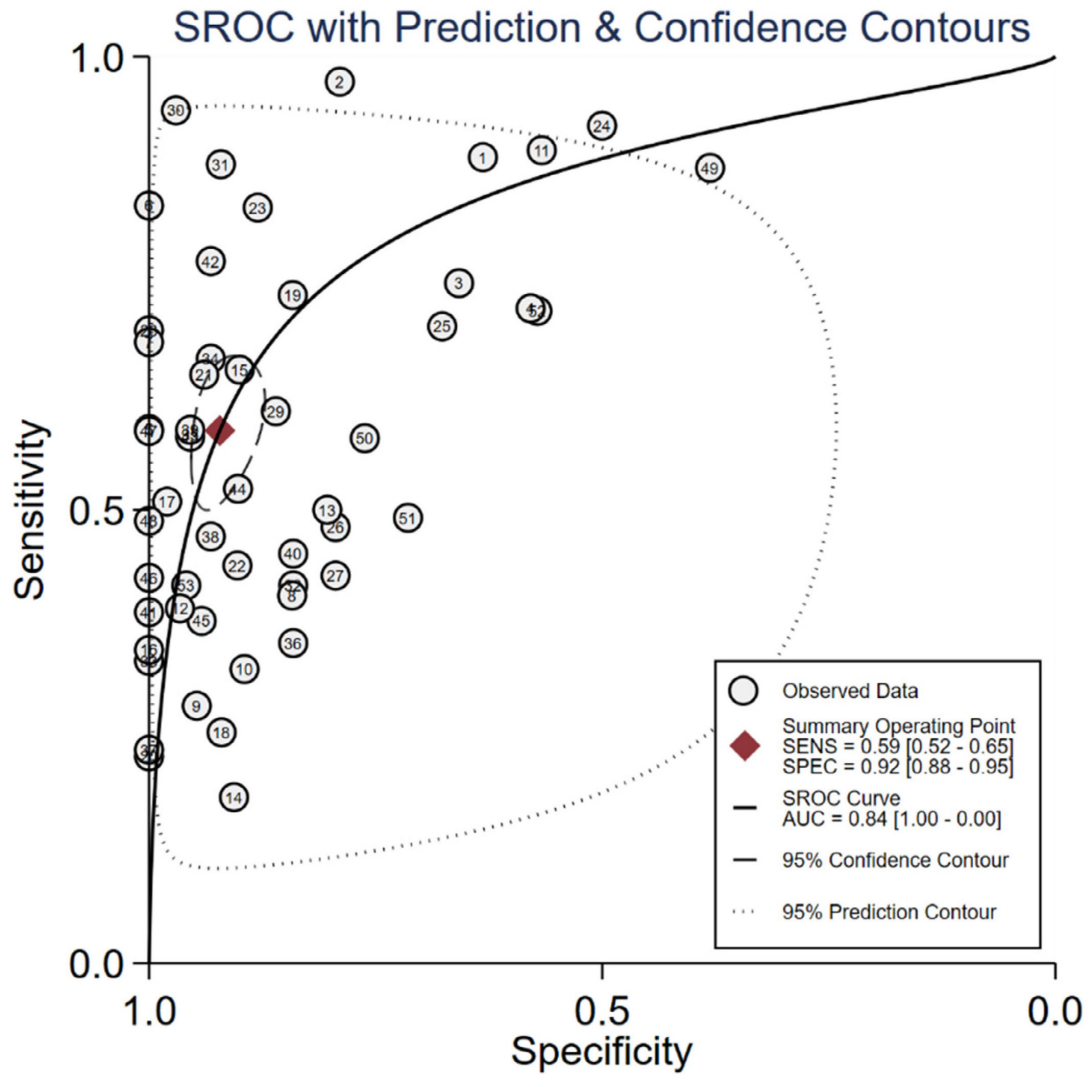
Summary of AUC of cfDNA markers for the diagnosis of gastric cancer.

We evaluated the diagnostic efficiency of cfRNAs and cfDNAs, as shown in Figure 4 and Figure 5, respectively. The meta results showed that the diagnostic accuracy of cfRNA markers (sensitivity 0.80, specificity 0.80, and AUC 0.87) was different from that of cfDNA markers (sensitivity 0.59, specificity 0.92, and AUC 0.84). Compared to cfDNAs, the diagnostic ability of cfRNAs was better, with sensitivity increasing from 0.59 to 0.80, but worse with specificity decreasing from 0.92 to 0.80. However, differences with respect to AUCs were very limited.

## 4. Discussion

Our systematic review identified a total of 84 cfRNAs and 32 cfDNAs from 79 eligible studies evaluating the diagnostic performance of circulating nucleic acids for GC detection. Forty-four studies integrated individual markers into panels. Only five studies 34, 35, 45, 46, 84 conducted stage-specific analysis for the diagnostic performance of cfRNAs and cfDNAs. However, due to the lack of sufficient data, stage-specific miRNA for GC is still elusive. Overall, cfRNA and cfDNA markers show favorable diagnostic performance for GC. Compared to cfDNA markers, cfRNA markers showed better sensitivity but worse specificity for GC detection.

Cell-free RNA (cfRNA) typically includes encoded mRNAs and non-coding RNAs such as lncRNAs, miRNAs, circRNAs, and piRNAs 91, 92. Aalami et al. 12 revealed a higher miR-223 expression (3.10-fold expression) in patients with GC compared to controls. Chen et al. 16 showed that miR-421 could achieve a satisfactory diagnostic efficiency in distinguishing GC patients from healthy controls with an AUC of 0.981 (sensitivity = 96.67% and specificity = 95.56%). In this study, they found that plasma miR-421 could well distinguish precancerous lesions of gastric cancer patients from healthy controls with an AUC of 0.872 (sensitivity = 66.29% and specificity = 95.56%). Furthermore, the diagnostic efficacy of miR-421 was markedly higher than traditional tumor markers, such as CA153, CA211, and CA50. A study by Chen et al. 15 indicated that a combination of plasma miR‑650 and CA211 was an effective and novel diagnostic biomarker panel in the diagnosis of GC. However, due to the instability of most extracellular free mRNAs and lncRNAs, they are not suitable as tumor markers. Therefore, researchers have focused more on stable small non-coding RNAs (ncRNAs), such as miRNAs, as diagnostic and prognostic markers for cancer in the past decade. Although miRNAs account for less than 2% of total ncRNAs, abnormal expression has been found in gastric tissue for pre-cancer events (such as Helicobacter pylori infection and pre-cancerous lesions including chronic atrophic gastritis and intestinal metaplasia), as well as in early and late-stage gastric cancer 93, 94. Therefore, many studies have proposed panels with higher sensitivity and specificity, which can better diagnose tumors and determine their prognosis than single miRNAs. In recent years, with advances in small non-coding RNA sequencing technology, more and more circRNAs and piRNAs have been discovered and named, becoming a hot topic in cancer research. Similar to miRNAs, circRNAs and piRNAs are also characterized by their abundance, stability, and tissue specificity, and they are widely circulated in various body fluids and extracellular vesicles 9, 92. Hence, circRNAs and piRNAs may become new diagnostic biomarkers for gastric cancer. Overall, small ncRNAs have certain advantages as tumor markers, as mentioned above. To better diagnose gastric cancer in its early stages, researchers should continue to explore more small non-coding RNAs and propose more accurate and specific diagnostic panels in the future.

cfDNA has become an effective biomarker for cancer detection. Pimson et al. 83 confirmed that the cfDNA level of GC patients was higher than that of healthy controls. The diagnostic sensitivity and specificity were 94% and 97% respectively. The cfDNA can also be used to distinguish benign gastric diseases (BGD) and early gastric diseases (EGC). Cao et al. 73 showed that SEPT9 was methylated in 28.4% (21/74) of (EGC) cases but in only 6.1% (6/99) of BGD cases (P<0.001), RNF180 was found to be methylated in 32.4% (24/74) of EGC cases, which was significantly higher than the 13.1% (13/99) of BGD cases (P<0.001). Lin et al. 80 discovered that the combined hypermethylated status of FAM5C and MYLK correlated with tumor size (P < 0.001), tumor invasion depth (P = 0.001) and tumor-node-metastasis (TNM) stage (P = 0.003). With the rapid decrease in sequencing costs and the emergence of more efficient library preparation techniques, researchers are able to detect cancer-related point mutations, copy number variations, and methylation markers at increasingly early disease stages 95-98. Although these methods hold promise for cancer screening, they are fundamentally limited by the amount of tumor DNA shed into the bloodstream during cell death 99. Small or slowly growing tumors release less DNA into circulation, resulting in decreased sensitivity for early cancer detection using cfDNA 100. In addition, most cfDNA features, such as small nucleotide variations, are not tumor tissue-specific, making it difficult to predict the tumor origin site for positive cancer screening patients. Recently, targeted analysis of methylation markers on cfDNA has been shown to detect and locate cancer with high specificity 101. Technological advances make cfDNA detection more specific, but recent reports indicate that aging individuals exhibit a background mutation landscape, including cancer driver genes 102, 103. White blood cells are the main source of this background, which requires measuring the clonal hematopoiesis of indeterminate potential (CHIP) mutations and eliminating these mutations from the overall pool of cfDNA aberrations detected in individual patients 104.

Despite the increasing amount of research on cell-free nucleic acids as tumor markers in recent years and the identification of many nucleic acid markers for cancer diagnosis, personalized treatment, and prognosis, their reproducibility is low and their application in clinical practice is limited. This situation is mainly caused by several factors. Firstly, the clinical sample sizes used in most studies are too small, and the heterogeneity of tumors affects the representativeness of identified nucleic acid markers. Therefore, to ensure the reliability and effectiveness of research, it is necessary to increase the number of research samples and fully consider the impact of tumor heterogeneity. Secondly, the clinical sample types collected in most studies are single, and the identified nucleic acid markers cannot accurately distinguish between cancer patients and those with related precursor lesions. Therefore, different stages of clinical samples should be studied to obtain more accurate nucleic acid markers and improve their application value. Finally, so far, there has been no unified standard for sample collection and detection methods. Differences in sample processing, detection methods, and data processing standards can all lead to different results. Therefore, a set of standardized sample collection and detection methods needs to be developed to ensure the consistency and comparability of data.

Sample preparation is an essential pre-analytical factor that affects the identification of potential marker candidates. In 1999, the World Health Organization (WHO) observed a change made by some laboratories that indicated plasma as the preferred sample for the analysis of blood extracellular constituents as it was more representative of the in-vivo status of the patient compared to serum 105. Plasma is recommended for metabolites in general, for circulating DNA and RNA associated with tumors in cell-free samples, and for mitochondrial RNA, while serum remains the preferred sample for proteomics and lipidology 106, 107. Since the concentrations of cellular miRNAs and cfDNAs are relatively high compared to those in plasma and serum, it is recommended to use a second high-speed centrifugation or filtration step during blood processing 108-111. This step serves to remove the potentially retained cells and cell debris from plasma or serum, minimizing the possibility of blood cell contamination that could lead to erroneous interpretation of results. However, only a few of the included studies applied such a high-speed centrifugation step (Table S1, S2). Hemolysis of samples is another factor that could cause variability in miRNA findings, as Pritchard et al 111. showed that hemolysis can alter plasma miRNA biomarker levels by up to 50-fold. 110 The diverse extraction and quantification methods used in the included studies could also lead to bias in marker identification. Another important yet unresolved issue in circulating RNA investigation is normalization. Considering the limitations mentioned above, further accurate studies on the use of cell-free nucleic acid as promising diagnostic biomarkers for GC are urgently needed.

## 5. Conclusions

Our review concluded that cell-free nucleic acids hold the potential to serve as diagnostic markers for GC. Given that cfRNA and cfDNA markers have demonstrated favorable diagnostic performance for GC, their combination may enhance diagnostic efficiency. However, considering the differences in marker identification caused by pre-analytical factors and analytical factors, future studies should focus on standardizing sample processing procedures and detection protocols.

## Supplementary Material

Supplementary figures and tables.

## Figures and Tables

**Table 1:** Diagnostic performance of individual cell-free RNA markers in gastric cancer

				Case VS Control									
Reference	Author	Year	Region	Number	Age (years)	Male (%)	cfRNA marker	Specimen	Stage	SEN (%)	SPE (%)	AUC	P-value
12	Aalami AH	2020	Iran	39/39	65/69	80/77	miR-223-5p	Serum	I-III	90	85	0.9	<0.0001
13	Bai SY	2019	china	50/53	/	74/NA	miR-551b-3p	Serum	I-IV	70	96	0.86	<0.001
14	Cai CC	2019	china	63/29	63/NA	71/NA	Exo-Lnc RNA PCSK2-2:1	Serum	I-IV	84	87	0.9	/
15	Chen JL	2020	china	90/45	65/59	76/47	miR‑650	Plasma	I-IV	62	93	0.7	0.0001
16	Chen JL	2019	china	90/45	/	/	miR-421	Plasma	I-IV	97	98	0.98	<0.0001
17	Chen SJ	2017	china	104/104	/	72/72	circ_0000190	Plasma	I-IV	72	68	0.75	/
18	Chen X	2020	china	80/80	/	/	miR-125b-5p	Serum	I-IV	78	60	0.68	<0.001
							miR-196a5p			70	70	0.73	<0.001
							miR-1-3p			80	60	0.72	<0.001
							miR-149-5p			58	68	0.66	<0.001
19	Dong ZG	2019	china	119/31	/	75/61	Exosomal MT1-MMP mRNA	Serum	I-IV	64	87	0.79	/
20	Elsayed ET	2018	Egypt	50/50	/	/	lncRNA HOTAIR	Plasma	I-IV	88	84	0.94	/
21	Emami SS	2019	Iran	30/30		63/67	miR-21	Plasma	I-IV	87	72	0.89	<0.0001
							miR-222			63	56	0.75	0.044
22	Fu ZC	2014	china	114/56	/	47/NA	miR-222	Plasma	I-IV	66	88	0.85	/
23	Gong Y	2018	china	42/60	/	64/62	miR-199a	Serum	I-IV	93	71	0.92	/
24	Gu XL	2021	china	130/110	/	72NA	tsr016141	Serum	I-IV	75	78	0.81	/
25	Guo YT	2020	china	90/90	/	67/NA	miR-296-5p	Serum	I-IV	84	92	0.92	<0.001
							miR-28-3p			91	80	0.91	<0.001
26	Han WW	2021	china	146/95	53/46	66/58	miR-135	Serum	I-IV	78	80	0.87	/
							miR-20a			79	72	0.79	/
27	Hou X	2015	china	80/80	68/67	58/55	miR-106a	Plasma	I-IV	94	76	0.89	/
29	Huang YJ	2021	china	111/89	/	63/NA	tRF-31-U5YKFN8DYDZDD	Serum		60	81	0.74	/
30	Ji B	2019	china	168/74	/	60/NA	lncRNA LINC00086	Plasma	I-IV	73	84	0.86	/
							miR-214			73	92	0.88	/
31	Kong S	2019	china	30/30	/	/	circ_0001821	Plasma	I-IV	87	87	0.87	/
32	Kong Y	2019	china	184/78	62/63	61/56	miR-25	Plasma	I-IV	89	85	0.77	<0.0001
33	Li BH	2017	china	116/85	61/58	66/55	miR-320	Plasma	I-IV	82	76	0.86	/
34	Li C	2013	china	180/80	58/59	69/65	miR-199a-3p	Plasma	I-IV	80	74	0.84	/
35	Li C	2013	china	80/70	57/59	69/64	miR-199a-3p	Plasma	I-IIA	76	74	0.82	/
36	Li FX	2017	china	65/65	54/56	77/77	miR-106	Plasma	I-IV	86	92	0.9	<0.001
							miR-93			82	74	0.76	<0.001
							miR-25			88	77	0.82	<0.001
37	Li Y	2019	china	40/40	58/60	65/55	miR-381	Serum	I-II	83	93	0.92	<0.0001
38	Liu H	2017	china	137/145	54/54	62/64	miR-217	Plasma	I-IV	81	83	0.89	/
39	Liu HF	2017	china	145/145	/	65/NA	miR-205	Serum	I-III	98	83	0.91	/
40	Liu HS	2012	china	40/41	/	/	miR-378	Serum	I-IV	88	71	0.86	/
41	Liu WW	2020	china	89/73	/	71/NA	lncRNA FEZF1-AS1	Serum	I-IV	75	66	0.81	<0.001
							lncRNA AFAP1-AS1			76	56	0.82	<0.001
42	Liu Y	2019	china	94/40	59/59	61/65	lncRNA HOXA11-AS	Serum	I-IV	79	98	0.92	0.001
43	Park JL	2015	Korea	35/35	52/49	51/51	miR-27a	Plasma	I-IV	77	56	0.7	/
44	Qin SY	2021	china	98/82	/	58/NA	LncRNA HCP5	Serum	I-IV	80	70	0.82	/
46	Saliminejad K	2022	Iran	97/100	59/56	64/NA	miR-18a	Plasma	I-IV	55	57	0.67	0.027
							miR-21			56	54	0.65	0.042
							miR-125b			64	65	0.69	0.004
47	Shan LC	2019	china	117/100	58/50	75/58	lncRNA UCA1	Serum	I-IV	93	79	0.76	/
48	Shao YF	2022	china	42/40	/	/	circ_0086720	Plasma	I-II	67	87	0.77	<0.001
49	Shen Y	2020	china	98/40	58/59	49/63	miR-30c	Serum	I-IV	90	84	0.92	/
50	Shen YJ	2021	china	89/98	/	67/NA	tRF-19-3L7L73JD	Plasma	I-IV	40	79	0.62	/
52	Sun XY	2022	china	71/60	/	69/38	circ_0002874	Plasma	I-IV	87	77	0.84	/
53	Tian WY	2022	china	112/40	58/NA	63/63	miR-181	Serum	I-II	84	78	0.82	/
							miR-652			86	80	0.84	/
54	Wu DY	2017	china	32/32	/	NA/67	miR-503	Serum	I-IV	97	79	0.89	0.006
55	Wu JH	2015	china	90/90	/	49/NA	miR-421	Serum	I-IV	90	86	0.78	/
56	Wu JH	2015	china	50/50	/	48/NA	miR-21	Serum	I-IV	88	80	0.91	/
57	Xiao K	2021	china	113/27	/	74/30	EV lncRNA CCAT1	Serum	I-IV	80	93	0.89	/
58	Yan JN	2022	china	62/46	/	70/NA	circ_0001020	plasma	I-IV	47	98	0.74	<0.001
59	Yin G	2020	china	80/60	58/53	56/42	circ_0141633	Serum	I-IV	85	94	0.84	/
60	Yuan RS	2016	china	48/22	/	79/NA	miR-106a	Plasma	I-IV	77	64	0.83	/
61	Zeng QH	2014	china	40/36	/	70/NA	miR-17	Serum	I-IV	81	88	0.88	/
							miR-106b			75	93	0.86	/
62	Zeng WW	2020	china	86/50	60/45	42/52	miR-101-3p	Serum	I-IV	72	86	0.87	<0.0001
63	Zhang WW	2021	china	100/80	/	61/NA	circ_0007507	Serum	I-IV	73	85	0.83	<0.001
64	Zhang Y	2022	china	124/119	/	67/NA	tRF-23-Q99P9P9NDD	Serum	I-IV	67	86	0.78	/
65	Zhao QF	2018	china	102/105	/	71/NA	circular RNA 0000181	Plasma	I-IV	21	99	0.58	/
66	Zheng GD	2021	china	168/50	61/40	70/NA	Exosomal miR-590-5p	Serum	I-IV	64	86	0.81	/
67	Zhou XY	2015	china	70/70	/	63/NA	lncRNA H19	Plasma	I-IV	83	73	0.84	/
68	Zhou XY	2015	china	50/50	/	/	miR-223	Plasma	I-IV	70	80	0.81	/
69	Zong W	2019	china	110/84	/	662/NA	lncRNA CTC-497E21.4	Serum	I-IV	82	75	0.85	/

SEN: sensitivity; SEP: specificity; AUC: area under the curve; NA: not available.

**Table 2:** Diagnostic performance of cell-free RNA panels in gastric cancer

				Case VS Control									
Reference	Author	Year	Region	Number	Age (years)	Male (%)	Panel	Specimen	Stage	SEN (%)	SPE (%)	AUC	P-value
18	Chen X	2020	china	80/80	/	/	Panel A	Serum	I-IV	86	79	0.89	<0.001
28	Huang SK	2016	china	62/59	/	/	Panel B	Serum	I-IV	86	98	0.92	/
41	Liu WW	2020	china	89/73	/	71/NA	Panel C	Serum	I-IV	85	60	0.87	<0.0001
45	Roy S	2022	Japan	102/48	/	/	Panel D	Serum	I-IV	89	62	0.83	/
				69/48	/	/			I-II	90	60	0.82	/
46	Saliminejad K	2022	Iran	97/100	59/56	64/NA	Panel E	Plasma	I-IV	86	85	0.92	<0.001
				31/100	NA/56	/			I-II	83	75	0.83	0.001
				59/100	NA/56	/			III-IV	86	75	0.93	0.001
51	So JBY	2021	Singaporean	125/4441	57/57	61/53	Panel F	Serum	I-IV	87	68	0.85	/
61	Zeng QH	2014	china	40/36	/	70/NA	Panel G	Serum	I-IV	83	87	0.91	/

SEN: sensitivity; SEP: specificity; AUC: area under the curve; NA: not available. Panel A: miR-125b-5p, miR-196a5p, miR-1-3p, miR-149-5p; Panel B: miR-21, miR-31, miR-92a, miR-181b, miR-203; Panel C: lncRNA FEZF1-AS1, lncRNA AFAP1-AS1; Panel D: circ_0045602,circ_0008768, circ_0007380, circ_0002019, circ_0006089, circ_0034398, circ_0052001, circ_0001013; Panel E: miR-18a, miR-21, miR-125b; Panel F: miR-140, miR-183, miR- 30e, miR- 103a, miR-126, miR-93, miR-142, miR-21, miR29c, miR-424,miR-340, miR- 181a; Panel G: miR-17, miR-106b.

**Table 3:** Diagnostic performance of individual cell-free DNA markers in gastric cancer

				Case VS Control											
Reference	Author	Year	Region	Number	Age (years)	Male (%)	Target gene	Alteration type	Method	Specimen	Stage	SEN (%)	SPE (%)	AUC	P-value
70	Anderson BW	2018	USA	36/38	/	61/58	ELMO1	methylation	QuARTS	Plasma	I-IV	96	78	0.94	/
							ZNF569					76	66	0.72	/
							C13orf18					73	58	0.73	/
71	Ioanna B	2013	Greece	73/20	67/64	70/NA	SOX17	methylation	MSP	Serum	I-IV	59	100	/	/
72	Ioanna B	2015	Greece	73/20	67/64	70/NA	APC	methylation	MSP	Serum	I-IV	84	100	/	/
							RASSF1A					68	100	/	/
73	Cao CQ	2020	China	74/57	/	64/46	SEPT9	methylation	MSP	Plasma	I-II	28	94	0.62	/
							RFP 180					32	90	0.64	/
74	Chen L	2012	China	58/30	62/55	/	FAM5C	hypermethylation	MSP	Serum	I-IV	/	/	0.64	/
							MYLK					/	/	0.82	/
75	Han J	2014	China	92/88	/	58/61	MINT2	methylation	MSP	Serum	I-IV	39	97	/	/
76	Hideura E	2020	Japan	50/61	72/58	82/52	RUNX3	methylation	digital PCR	Serum	I-II	50	80	0.7	/
77	Lee HS	2013	Korea	153/96	/	59/NA	SEPT9	methylation	MSP	Plasma	I-IV	18	91	/	/
78	Li H	2022	China	55/50	66/55	73/52	KCNQ5	methylation	MSP	Plasma	I-IV	35	100	0.69	/
							C9orf50					51	98	0.74	/
							CLIP4					25	92	0.6	/
				57/82	66/28	67/30	KCNQ5					23	100	0.63	/
							C9orf50					65	94	0.82	/
							CLIP4					44	90	0.68	/
80	Lin ZH	2017	China	131/34	61/57	67/65	ZIC1	methylation	MSP	Plasma	I-IV	70	69	/	/
							HOXD10					48	80	/	/
							RUNX3					43	79	/	/
81	Ling ZQ	2013	China	202/88	59/NA	/	XAF1	methylation	MSP	Serum	I-IV	70	100	/	/
82	Miao J	2020	China	92/50	60/NA	73/NA	SFRP2	methylation	MSP	Plasma	I-IV	61	86	0.78	/
83	Pimson C	2016	Thailand	101/202	/	42/NA	PCDH10	methylation	MSP	Plasma	I-IV	94	97	/	/
							RASSF1A					88	92	/	/
84	Saliminejad K	2020	Iran	96/88	/	/	P16	methylation	MSP	Plasma	I-IV	42	84	0.63	<0.001
							RASSF1A					33	100	0.67	<0.001
							RPRM					67	93	0.8	<0.001
							RUNX3					58	95	0.77	<0.001
				34/88	/	/	P16				I-II	35	84	0.6	0.026
							RASSF1A					24	100	0.62	<0.001
							RPRM					47	93	0.7	<0.001
							RUNX3					59	95	0.77	<0.001
				62/88	/	/	P16				III-IV	45	84	0.65	<0.001
							RASSF1A					39	100	0.69	<0.001
							RPRM					77	93	0.85	<0.001
							RUNX3					58	95	0.76	<0.001
85	Xu JB	2021	China	151/224	/	75/51	SEPT9	methylation	MSP	Plasma	I-IV	52	90	0.77	/
							RNF180					38	94	0.72	/
86	Yang QF	2013	China	40/22	/	83/NA	BCL6B	hypermethylation	MSP	Plasma	I-IV	43	100	/	/
87	Yu JL	2014	China	92/88	/	59/61	TIMP-3	methylation	MSP	Serum	I-IV	59	100	/	/
88	Zhang H	2014	China	41/21	/	73/NA	Spastic paraplegia-20	hypermethylation	MSP	Plasma	I-IV	49	100	/	/
89	Zhang X	2014	China	57/42	61/57	68/64	RNF180	methylation	MSP	Plasma	I-IV	58	76	/	/
							DAPK1					49	71	/	/
							SFRP2					72	57	/	/
90	Zhao LY	2022	China	60/122	/	73/NA	SEPT9	methylation	MSP	Plasma	I-IV	42	96	0.7	/

SEN: sensitivity; SEP: specificity; AUC: area under the curve; NA: not available; MSP: methylation specific PCR; QuARTS: Quantitative allele-specific real-time target and signal amplification.

**Table 4:** Diagnostic performance of cell-free DNA panels in gastric cancer

				Case VS Control										
Reference	Author	Year	Region	Number	Age (years)	Male (%)	Panel	Alteration type	Method	Specimen	Stage	SEN (%)	SPE (%)	AUC
70	Anderson BW	2018	USA	36/38	/	61/58	Panel A	methylation	QuARTS	Plasma	I-IV	90	63	0.91
73	Cao CQ	2020	China	74/57	/	64/46	Panel B	methylation	MSP	Plasma	I-II	41	85	0.65
74	Chen L	2012	China	58/30	62/55	/	Panel C	hypermethylation	MSP	Serum	I-IV	90	58	0.84
78	Li H	2022	China	55/50	66/55	73/52	Panel D	methylation	MSP	Plasma	I-IV	66	90	0.81
				57/82	66/28	67/30						74	84	0.85
79	Li WH	2016	China	48/25	57/53	81/72	Panel E	hypermethylation	MSP	Serum	I-IV	83	88	/
80	Lin ZH	2017	China	131/34	61/57	67/65	Panel F	methylation	MSP	Plasma	I-IV	92	50	/
89	Zhang X	2014	China	57/42	61/57	68/64	Panel G	methylation	MSP	Plasma	I-IV	87	38	/

SEN: sensitivity; SEP: specificity; AUC: area under the curve; MSP: methylation-specific PCR; QuARTS: Quantitative allele-specific real-time target and signal amplification. Panel A: ELMO1, ZNF569, C13orf18; Panel B: Septin 9, RFP 180; Panel C: FAM5C, MYLK; Panel D: KCNQ5, C9orf50, CLIP4; Panel E: OSR2, VAV3, PPFIA3; Panel F: ZIC1, HOXD10, RUNX3; Panel G: RNF180, DAPK1, SFRP2.
