# Supplementary material for: Cell-free Nucleic Acid as Promising Diagnostic Biomarkers for Gastric Cancer: a Systematic Review
Source: J Cancer. 2024 Mar 25;15(10):2900–12. doi: 10.7150/jca.92704 (PMC11064260; doi:10.7150/jca.92704)
Supplement: Supplementary file 1 — Supplementary figures and tables. [file jcav15p2900s1.pdf]

Supplemental Material

Cell-free Nucleic Acid as Promising Diagnostic Biomarkers for Gastric Cancer: a Systematic Review

Qun Zhang #, Zhouyuan Du #, Xiaoxiao Wang, Fen Li, Yi Liu, Jingjing Sun, Lin Zhang, Yong Xiao, Xiaoming Lu, Haixin Yu*, Tao Liu*

Affiliations
Department of Digestive Surgical Oncology, Union Hospital, Tongji Medical College, Huazhong University of Science and Technology, Wuhan 430022, China

Contents:

Table S1, pages 2-3

Table S2, page 4

Table S3, page 5

Table S4, page 6

Figure S1, page 6

Figure S2, page 7

Figure S3, page 8

Figure S4, page 8

Reference, pages 9-13

Table S1. Protocols of blood RNA detection.

Reference	Author	Year	Specimen	Centrifugation	Extraction	Normalization
1	Aalami AH	2020	Serum	1500rpm for 10min, 12000rpm for 2min	Plasma/Serum RNA Purification Mini RNA Kit	snord47
2	Bai SY	2019	Serum	NA	TRIzol reagent	miR-16
3	Cai CC	2019	Serum	3000g for 10 min	HiPure Exosome kits	β-actin
4	Chen JL	2020	Plasma	1520g for 5 min	Blood MicroRNAExtraction and Purification kit	miR-16
5	Chen JL	2019	Plasma	3000 rpm for 5min	NA	miR-16
6	Chen SJ	2017	Plasma	NA	TRIzol LS Reagent	GAPDH
7	Chen X	2020	Serum	1000g for 10 min, 15000g for 5 min	TRIzol LS isolation kit	miR-39
8	Dong ZG	2019	Serum	3000g for 10 min	exoRNeasy Serum/Plasma Midi Kit	GAPDH
9	Elsayed ET	2018	Plasma	1200g for 10 min, 12000g for 10 min	miRNeasy Mini Kit	GAPDH
10	Emami SS	2019	Plasma	1500g for 15 min, 10000g for 5 min	Qiagen miRNeasy Serum/Plasma Kit	miR-39
11	Fu ZC	2014	Plasma	1200g for 10 min, 12000g for 10 min	mirVana PARIS kit	U6 snRNA
12	Gong Y	2018	Serum	3500rpm for 10 min	MiRcute miRNA extraction kit	NA
13	Gu XL	2021	Serum	NA	Total RNA Pure and Isolation Kit with Spin Column	U6 snRNA
14	Guo YT	2020	Serum	3000g for 15 min	TRIzol reagent	U6 snRNA
15	Han WW	2021	Serum	3500rpm for 10 min	Serum microRNA rapid extraction kit	U6 snRNA
16	Hou X	2015	Plasma	2000g for 10 min, 12000g for 10 min	Trizol kit	U6 snRNA
17	Huang SK	2016	Serum	820g for 10 min, 16000g for 10 min	Trizol LS Reagent	miR-16
18	Huang YJ	2021	Serum	NA	Total RNA Pure and Isolation Kit with Spin Column	U6 snRNA
19	Ji B	2019	Plasma	1500g for 10 min, 12000g for 10 min	TRIzol reagent	U6 snRNA
20	Kong S	2019	Plasma	NA	TRIzol reagent	GAPDH
21	Kong Y	2019	Plasma	1500rpm for 30 min, 3000rpm for 5 min, 4500rpm for 5 min	NA	U6 snRNA
22	Li BH	2017	Plasma	1500rpm for 10 min	TRIzol reagent	U6 snRNA
23	Li C	2013	Plasma	1000rpm for 10 min, 15000rpm for 10 min	mirVanaTM PARISTM Kit	U6 snRNA
24	Li C	2013	Plasma	1000rpm for 10 min, 15000rpm for 10 min	mirVanaTM PARISTM Kit	U6 snRNA
25	Li FX	2017	plasma	2000g for 30 min, 4000g for 5 min, 8000g for 5 min	NA	U6 snRNA
26	Li Y	2019	Serum	NA	miRcute Serum/Plasma miRNA Isolation Kits	U6 snRNA
27	Liu H	2017	Plasma	3000rpm for 10 min	QIAGEN miRNeasy Mini Kit	U6 snRNA
28	Liu HF	2017	Serum	3000g for 15 min	NA	NA
29	Liu HS	2012	Serum	820g for 10 min, 16000g for 10 min	mirVana miRNA isolation kit	U6 snRNA
30	Liu WW	2020	Serum	3000g for 10 min	Ambion mirVana PARIS kit	18S rRNA
31	Liu Y	2019	Serum	1200g for 10 min, 10000g for 10 min	Qiagen miRNeasy Serum/Plasma Kit	GAPDH
32	Park JL	2015	Plasma	2000g for 10 min	Ambion PARIS Kit	U6 snRNA
33	Qin SY	2021	Serum	NA	serum extraction kit	18S rRNA
34	Roy S	2022	Serum	NA	NA	β-actin
35	Saliminejad K	2022	Plasma	3000g for 10 min	RiboExTM LS reagent	U6 snRNA
36	Shan LC	2019	Serum	3500g for 10 min, 16000g for 10 min	Trizol LS Reagent	GAPDH

Table S1. Cont.

Reference	Author	Year	Specimen	Centrifugation	Extraction	Normalization
37	Shao YF	2022	Plasma	NA	NA	GAPDH
38	Shen Y	2020	Serum	3000rpm for 10 min	NA	NA
39	Shen YJ	2021	Plasma	NA	TRIzol LS	U6 snRNA
40	So JBY	2021	Serum	3000rpm for 10 min	miRNeasy serum/plasma miRNA isolation kit	NA
41	Sun XY	2022	Plasma	3000rpm for 10 min	RNA reagent kits	18S rRNA
42	Tian WY	2022	Serum	NA	TRIzol LS	U6 snRNA
43	Wu DY	2017	Serum	3000rpm for 30 min	BioTeKe miRNA extraction kit	U6 snRNA
44	Wu JH	2015	Serum	1000g for 10 min	SYBR Premix Ex Taq kit	U6 snRNA
45	Wu JH	2015	Serum	800g for 30 min	Trizol reagent	U6 snRNA
46	Xiao K	2021	Serum	3000g for 10 min	exoRNeasy Serum/Plasma Midi kit	GAPDH
47	Yan JN	2022	plasma	NA	TRIzol LS reagents	GAPDH
48	Yin G	2020	Serum	3500rpm for 10 min	RNAVzol LS	GAPDH
49	Yuan RS	2016	Plasma	3000rpm for 10 min	Trizol reagent	U6 snRNA
50	Zeng QH	2014	Serum	2000rpm for 10 min, 3000rpm for 5 min, 4500rpm for 5 min	RNA extraction kit	NA
51	Zeng WW	2020	Serum	1500rpm for 30 min, 3000rpm for 5 min, 4500rpm for 5 min	mirVana PARIS kit	U6 snRNA
52	Zhang WW	2021	Serum	NA	TRIzol LS reagent	GAPDH
53	Zhang Y	2022	Serum	NA	RNA purifcation kit	U6 snRNA
54	Zhao QF	2018	Plasma	NA	TRIzol LS Reagent	GAPDH
55	Zheng GD	2021	Serum	3000rpm for 10 min	Total Exosome RNA and Protein Isolation Kit	miR-39
56	Zhou XY	2015	Plasma	1500rpm for 30 min, 3000rpm for 5 min, 4500rpm for 5 min	Trizol Reagent	GAPDH
57	Zhou XY	2015	Plasma	NA	miRNeasy Mini kits	U6 snRNA
58	Zong W	2019	Serum	NA	serum extract Kit	18S rRNA

NA: not available

Table S2. Protocols of blood DNA detection.

Reference	Author	Year	Specimen	Centrifugation	Extraction
59	Anderson BW	2018	Plasma	NA	NA
60	Ioanna B	2013	Serum	3000rpm for 10min	High Pure Viral Nucleic Acid Kit
61	Ioanna B	2015	Serum	3000rpm for 10min	High Pure Viral Nucleic Acid Kit
62	Cao CQ	2020	Plasma	NA	plasma processing kit by BioChain
63	Chen L	2012	Serum	NA	QIAamp Circulating Nucleic Acid Kit
64	Han J	2014	Serum	1500rpm for 10min	QIAamp Circulating Nucleic Acid Kit
65	Hideura E	2020	Serum	NA	MagNA Pure Compact Nucleic Acid Isolation Kit
66	Lee HS	2013	Plasma	1500rpm for 10min, 1500g for 10min	Abbott m2000sp Sample Preparation System
67	Li H	2022	Plasma	NA	DNeasy Blood & Tissue Kit
68	Li WH	2016	Serum	NA	TIANamp Blood DNA Kit
69	Lin ZH	2017	Plasma	2500rpm for 15min	QIAamp DNA Blood Mini Kit
70	Ling ZQ	2013	Serum	NA	AxyPrep Mag Blood gDNA kit
71	Miao J	2020	Plasma	NA	DNeasy Blood & Tissue Kit
72	Pimson C	2016	Plasma	2000g for 15min	Genomic DNA Mini Kit
73	Saliminejad K	2020	Plasma	3000g for 10min	QIAamp Circulating Nucleic Acid Kit
74	Xu JB	2021	Plasma	NA	QIAamp Circulating Nucleic Acid Kit
75	Yang QF	2013	Plasma	1600g for 10min, 16000g for 10min	phenol/chloroform method
76	Yu JL	2014	Serum	1500rpm for 10min	QIAamp DNA Blood Mini Kit
77	Zhang H	2014	Plasma	NA	TIANamp Blood DNA Kit
78	Zhang X	2014	Plasma	1000g for 10min	QIAamp DNA Blood mini kit
79	Zhao LY	2022	Plasma	1350±150rpm for 10min (twice)	nucleic acid extraction kit

NA: not available

Table S3. Summary of studies reporting diagnostic performance of miRNAs for gastric cancer (only miRNAs that have been reported in >=2 studies are shown).

Study	miR-421	miR-21	miR-222	miR-106a	miR-25	miR-199a-3p	miR-93
Chen, 2019[5]	△↑						
Emami, 2019[10]		△↑	△↑				
Fu, 2014[11]			△↑				
Hou, 2015[16]				△↑			
Huang, 2016[17]		○↑					
Kong, 2019[21]					△↑		
Li, 2013[23]						△↑	
Li, 2013[24]						△↑	
Li, 2017[25]					△↑		△↑
Saliminejad, 2022[35]		○↑					
So, 2021[40]		○↑					○↑
Wu,2015[44]	△↑						
Wu,2015[45]		△↑					
Yuan,2016[49]				△↑			
Number Of Studies	2	5	2	2	2	2	2

○ represents miRNAs which are part of a panel; △ represents miRNAs which have only been analyzed individually and not as a part of a miRNA panel; ↑ represents up-regulation.

Table S4. Summary of studies reporting diagnostic performance of miRNAs for gastric cancer (only miRNAs that have been reported in >=2 studies are shown).

Study	RASSF1A	SEPT9	RUNX3	SFRP2	RNF180
Ioanna, 2015[61]	△				
Cao, 2020[62]		○			
Hideura, 2020[65]			△		
Lee, 2013[66]		△			
Lin, 2017[69]			○		
Miao, 2020[71]				△	
Pimson, 2016[72]	△				
Saliminejad, 2020[73]	△				
Xu, 2021[74]		△			△
Zhang, 2014[78]				○	○
Zhao,2022[79]		△			
Number Of Studies	3	4	2	2	2

○ represents DNAs which are part of a panel; △ represents DNAs which have only been analyzed individually and not as a part of a miRNA panel.

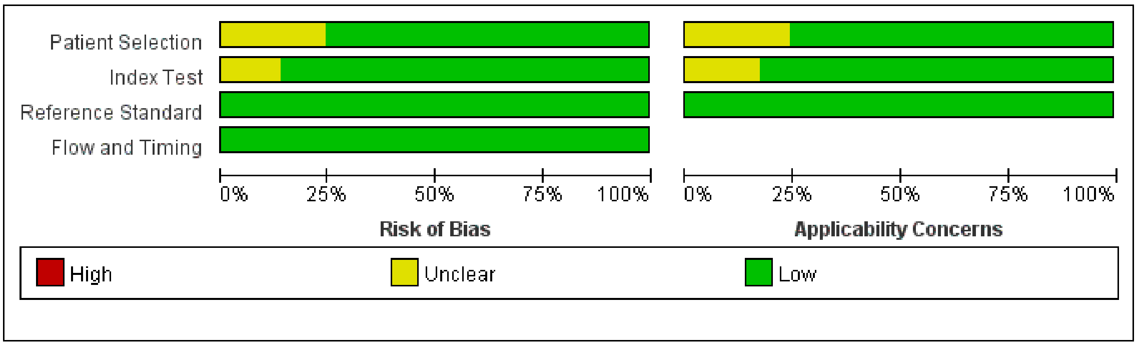

Figure S1: Risk of bias and applicability concerns graph: review authors' judgements about each domain presented as percentages across included studies. (cfRNA markers)

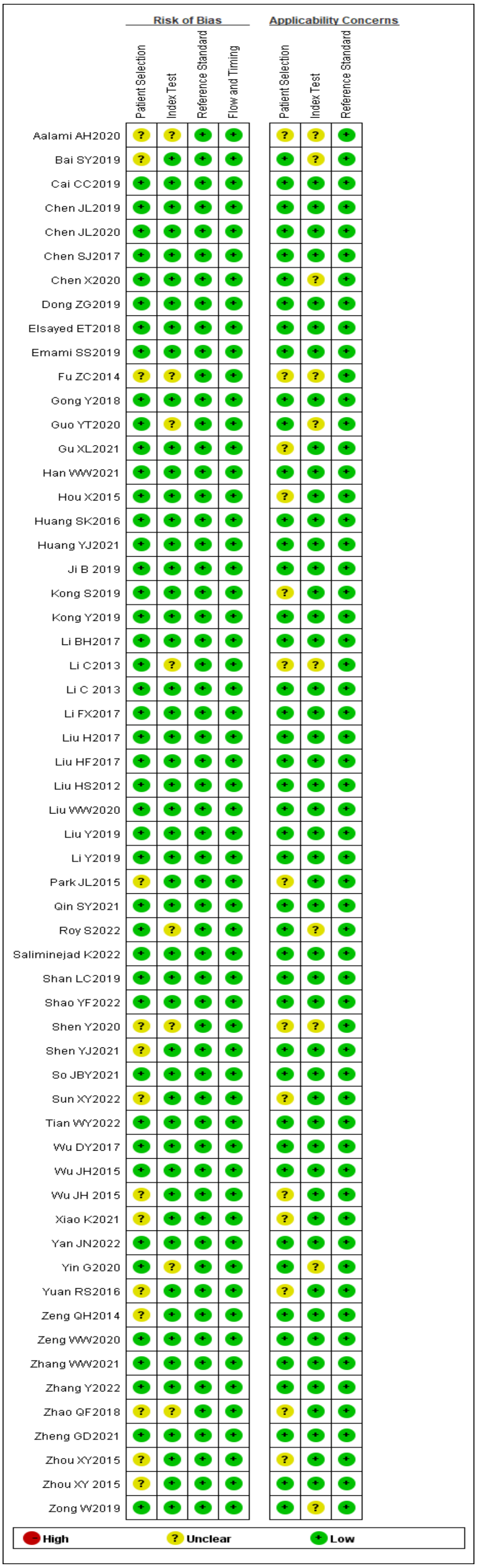

Figure S2: Risk of bias and applicability concerns summary: review authors' judgements about each domain for each included study. (cfRNA markers)

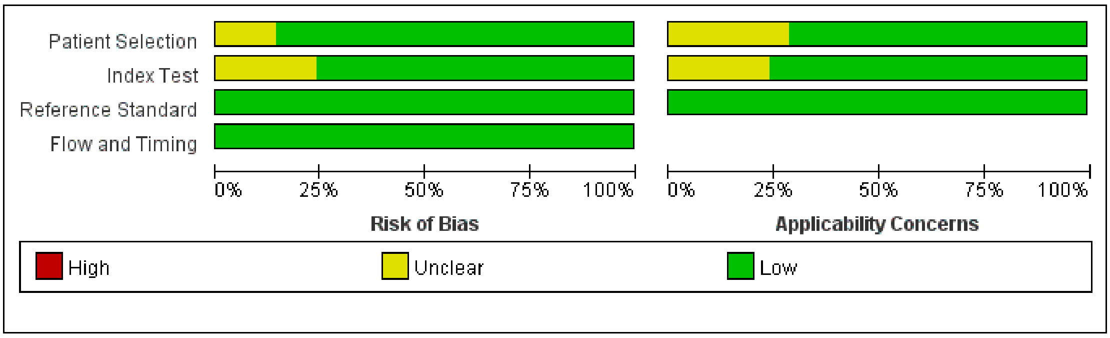

Figure S3: Risk of bias and applicability concerns graph: review authors' judgements about each domain presented as percentages across included studies. (cfDNA markers)

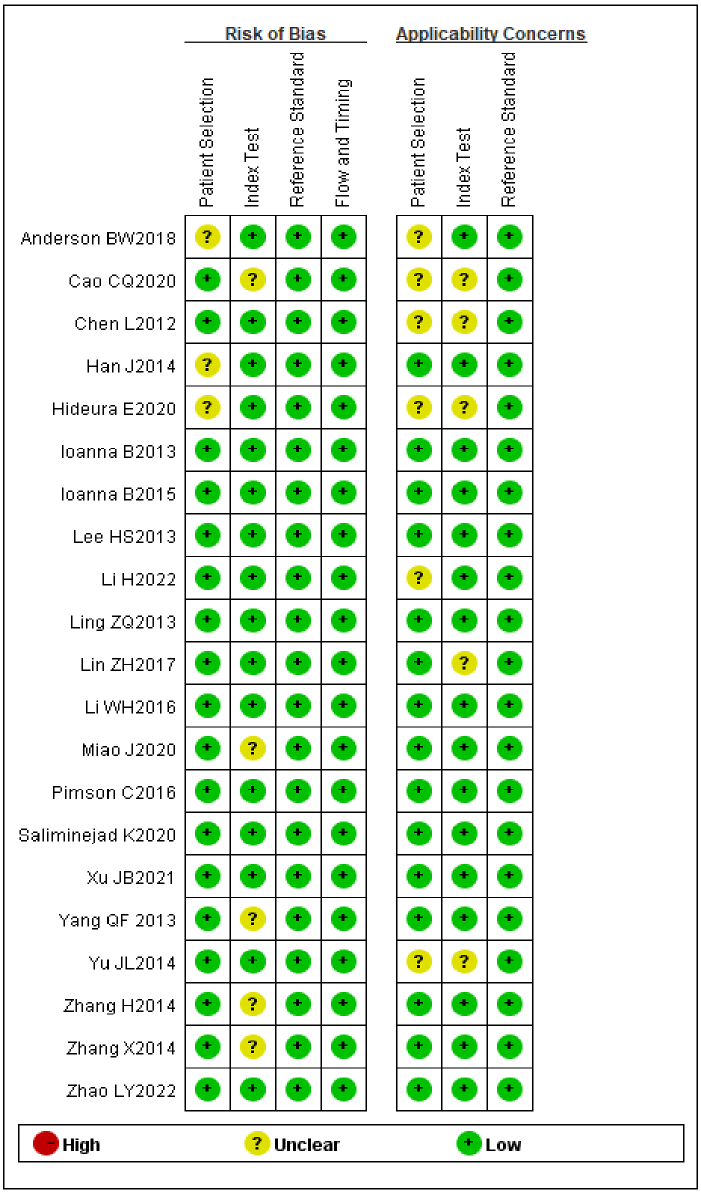

Figure S4: Risk of bias and applicability concerns summary: review authors' judgements about each domain for each included study. (cfDNA markers)

References:

[1]. Aalami, A.H., et al., Evaluation of the Diagnostic Properties of Serum hsa-miR-223-5p in the Detection of Gastric Cancer: A Case-Control Study. Anti-cancer agents in medicinal chemistry, 2020. 20(7): p. 800.

[2]. Bai, S., et al., Serum miR-551b-3p is a potential diagnostic biomarker for gastric cancer. The Turkish Journal of Gastroenterology, 2019. 30(5): p. 415-419.

[3]. Cai, C., et al., Serum Exosomal Long Noncoding RNA pcsk2-2:1 As A Potential Novel Diagnostic Biomarker For Gastric Cancer. Onco Targets Ther, 2019. 12: p. 10035-10041.

[4]. Chen, J., et al., Diagnostic value and clinical significance of circulating miR-650 and CA211 in detecting of gastric carcinoma. Oncol Lett, 2020. 20(5): p. 254.

[5]. Chen, J., et al., Mir-421 in plasma as a potential diagnostic biomarker for precancerous gastric lesions and early gastric cancer. PeerJ, 2019. 7: p. e7002.

[6]. Chen, S., et al., Using circular RNA hsa_circ_0000190 as a new biomarker in the diagnosis of gastric cancer. Clinica Chimica Acta, 2017. 466: p. 167-171.

[7]. Chen, X., et al., Use of a Four-miRNA Panel as a Biomarker for the Diagnosis of Stomach Adenocarcinoma. Disease Markers, 2020. 2020: p. 1-12.

[8]. Dong, Z., et al., Serum Membrane Type 1-Matrix Metalloproteinase (MT1-MMP) mRNA Protected by Exosomes as a Potential Biomarker for Gastric Cancer. Medical Science Monitor, 2019. 25: p. 7770-7783.

[9]. Elsayed, E.T., et al., Plasma long non-coding RNAHOTAIR as a potential biomarker for gastric cancer. The International Journal of Biological Markers, 2018. 33(4): p. 528-533.

[10]. Emami, S.S., et al., Evaluation of circulating miR-21 and miR-222 as diagnostic biomarkers for gastric cancer. J Cancer Res Ther, 2019. 15(1): p. 115-119.

[11]. Fu, Z., et al., Circulating miR-222 in plasma and its potential diagnostic and prognostic value in gastric cancer. Medical Oncology, 2014. 31(9).

[12]. Gong, Y., et al., Expression of miR-199a in gastric cancer tissues and serum and its clinical significance. INTERNATIONAL JOURNAL OF CLINICAL AND EXPERIMENTAL MEDICINE, 2018. 11(8): p. 7751-7760.

[13]. Gu, X., et al., Serum hsa_tsr016141 as a Kind of tRNA-Derived Fragments Is a Novel Biomarker in Gastric Cancer. Frontiers in Oncology, 2021. 11.

[14]. Guo, Y., et al., Diagnostic and Prognostic Value of Serum miR-296-5p and miR-28-3p in Human Gastric Cancer. Cancer Biotherapy and Radiopharmaceuticals, 2020.

[15]. Han, W., et al., Clinical value of miR-135 and miR-20a combined with multi-detector computed tomography in the diagnosis of gastric cancer. World Journal of Surgical Oncology, 2021. 19(1).

[16]. Hou, X., M. Zhang and H. Qiao, Diagnostic significance of miR-106a in gastric cancer. Int J Clin Exp Pathol, 2015. 8(10): p. 13096-101.

[17]. Huang, S., et al., Serum microRNA expression profile as a diagnostic panel for gastric cancer. Japanese Journal of Clinical Oncology, 2016. 46(9): p. 811-818.
[18]. Huang, Y., et al., Elucidating the Role of Serum tRF-31-U5YKFN8DYDZDD as a Novel Diagnostic Biomarker in Gastric Cancer (GC). Frontiers in Oncology, 2021. 11.
[19]. Ji, B., et al., Potential diagnostic and prognostic value of plasma long noncoding RNA LINC00086 and miR-214 expression in gastric cancer. Cancer Biomarkers, 2019. 24(2): p. 249-255.
[20]. Kong, S., et al., Identification of hsa_circ_0001821 as a Novel Diagnostic Biomarker in Gastric Cancer via Comprehensive Circular RNA Profiling. Frontiers in Genetics, 2019. 10.
[21]. Kong, Y., et al., Clinical significance of serum miR-25 as a diagnostic and prognostic biomarker in human gastric cancer. Cancer Biomarkers, 2019. 24(4): p. 477-483.
[22]. Li, B. and H. Zhang, Plasma microRNA-320 is a potential diagnostic and prognostic bio-marker in gastric cancer. Int J Clin Exp Pathol, 2017. 10(7): p. 7356-7361.
[23]. Li, C., et al., MiRNA-199a-3p in Plasma as a Potential Diagnostic Biomarker for Gastric Cancer. Annals of Surgical Oncology, 2013. 20(S3): p. 397-405.
[24]. Li, C., et al., MiRNA-199a-3p: A potential circulating diagnostic biomarker for early gastric cancer. Journal of Surgical Oncology, 2013. 108(2): p. 89-92.
[25]. Li, F., et al., The significance of elevated plasma expression of microRNA 106b~25 clusters in gastric cancer. PLOS ONE, 2017. 12(5): p. e0178427.
[26]. Li, Y., et al., Serum microRNA-381: A Potential Marker for Early Diagnosis of Gastric Cancer. Yonsei Medical Journal, 2019. 60(8): p. 720.
[27]. Liu, H., et al., MicroRNA-217 in plasma: a potential biomarker in gastric cancer. INTERNATIONAL JOURNAL OF CLINICAL AND EXPERIMENTAL MEDICINE, 2017. 10(2): p. 3313-3320.
[28]. Liu, H.F., et al., Downregulation of serum miR-205 as a potential biomarker for gastric cancer diagnosis, prognosis, and chemosensitivity prediction. INTERNATIONAL JOURNAL OF CLINICAL AND EXPERIMENTAL MEDICINE, 2017. 10(9): p. 13426-13432.
[29]. Liu, H., et al., Genome-wide microRNA profiles identify miR-378 as a serum biomarker for early detection of gastric cancer. Cancer Letters, 2012. 316(2): p. 196-203.
[30]. Liu, W., et al., Circulatinglong non-coding RNA FEZF1-AS1 and AFAP1-AS1 serve as potential diagnostic biomarkers for gastric cancer. Pathology - Research and Practice, 2020. 216(1): p. 152757.
[31]. Liu, Y., et al., Long noncoding RNA HOXA11-AS promotes gastric cancer cell proliferation and invasion via SRSF1 and functions as a biomarker in gastric cancer. World J Gastroenterol, 2019. 25(22): p. 2763-2775.
[32]. Park, J., et al., Cell-Free miR-27a, a Potential Diagnostic and Prognostic Biomarker for Gastric Cancer. Genomics & Informatics, 2015. 13(3): p. 70.

[33]. Qin, S., et al., LncRNA HCP5 : A Potential Biomarker for Diagnosing Gastric Cancer. Frontiers in Oncology, 2021. 11.

[34]. Roy, S., et al., Diagnostic efficacy of circular RNAs as noninvasive, liquid biopsy biomarkers for early detection of gastric cancer. Molecular Cancer, 2022. 21(1).

[35]. Saliminejad, K., et al., A Panel of Circulating microRNAs as a Potential Biomarker for the Early Detection of Gastric Cancer. Avicenna Journal of Medical Biotechnology, 2022.

[36]. Shan, L., C. Liu and C. Ma, High Expression of Serum UCA1 may be a Potential Biomarker for Clinical Diagnosis of Gastric Cancer. Clin Lab, 2019. 65(9).

[37]. Shao, Y., et al., Biological and clinical implications of hsa_circ_0086720 in gastric cancer and its clinical application. Journal of Clinical Laboratory Analysis, 2022. 36(5).

[38]. Shen, Y., et al., VALUE OF SERUM GASTRIC CANCER-ASSOCIATED ANTIGEN, MIR-30C, AND GASTRIN 17 IN THE EARLY DIAGNOSIS OF GASTRIC CANCER. ACTA MEDICA MEDITERRANEA, 2020. 36(3): p. 1393-1397.

[39]. Shen, Y., et al., Clinical diagnostic values of transfer RNA-derived fragment tRF-19-3L7L73JD and its effects on the growth of gastric cancer cells. Journal of Cancer, 2021. 12(11): p. 3230-3238.

[40]. So, J.B.Y., et al., Development and validation of a serum microRNA biomarker panel for detecting gastric cancer in a high-risk population. Gut, 2021. 70(5): p. 829-837.

[41]. Sun, X., et al., Diagnostic Value of Circular RNA hsa_circ_0002874 Expression in Peripheral Blood of Patients with Gastric Cancer. Lab Med, 2022. 53(1): p. 65-70.

[42]. Tian, W., X. Pang and F. Luan, Diagnosis value of miR‐181, miR‐652, and CA72‐4 for gastric cancer. Journal of Clinical Laboratory Analysis, 2022. 36(6).

[43]. Wu, D., et al., Decreased miR-503 expression in gastric cancer is inversely correlated with serum carcinoembryonic antigen and acts as a potential prognostic and diagnostic biomarker. Onco Targets Ther, 2017. 10: p. 129-135.

[44]. Wu, J., et al., MicroRNA-421 is a new potential diagnosis biomarker with higher sensitivity and specificity than carcinoembryonic antigen and cancer antigen 125 in gastric cancer. Biomarkers, 2014. 20(1): p. 58-63.

[45]. Wu, J., et al., Circulating MicroRNA-21 Is a Potential Diagnostic Biomarker in Gastric Cancer. Disease Markers, 2015. 2015: p. 1-8.

[46]. Xiao, K., et al., Clinical value of lncRNA CCAT1 in serum extracellular vesicles as a potential biomarker for gastric cancer. Oncol Lett, 2021. 21(6): p. 447.

[47]. Yan, J., et al., Hsa_circ_0001020 Serves as a Potential Biomarker for Gastric Cancer Screening and Prognosis. Digestive Diseases and Sciences, 2022. 67(8): p. 3753-3762.

[48]. Yin, G., et al., Circulating circRNA Has_Circ_0141633 Serves as a Potential Biomarker for Gastric Cancer. Clin Lab, 2020. 66(9).

[49]. Yuan, R., et al., Up-regulated Circulating miR-106a by DNA Methylation Promised a Potential Diagnostic and Prognostic Marker for Gastric Cancer. Anticancer Agents Med Chem, 2016. 16(9): p. 1093-100.
[50]. Zeng, Q., et al., Downregulation of serum miR-17 and miR-106b levels in gastric cancer and benign gastric diseases. Chin J Cancer Res, 2014. 26(6): p. 711-6.
[51]. Zeng, W., et al., Serum miR-101-3p combined with pepsinogen contributes to the early diagnosis of gastric cancer. BMC Medical Genetics, 2020. 21(1).
[52]. Zhang, W., et al., Circular RNA hsa_circ_0007507 May Serve as a Biomarker for the Diagnosis and Prognosis of Gastric Cancer. Frontiers in Oncology, 2021. 11.
[53]. Zhang, Y., et al., Evaluation of serum tRF-23-Q99P9P9NDD as a potential biomarker for the clinical diagnosis of gastric cancer. Molecular Medicine, 2022. 28(1).
[54]. Zhao, Q., et al., Clinical values of circular RNA 0000181 in the screening of gastric cancer. Journal of Clinical Laboratory Analysis, 2018. 32(4): p. e22333.
[55]. Zheng, G., et al., Exosomal miR-590-5p in Serum as a Biomarker for the Diagnosis and Prognosis of Gastric Cancer. Frontiers in Molecular Biosciences, 2021. 8.
[56]. Zhou, X., et al., Identification of the long non-coding RNA H19 in plasma as a novel biomarker for diagnosis of gastric cancer. Scientific Reports, 2015. 5(1).
[57]. Zhou, X., et al., Clinical role of circulating miR-223 as a novel biomarker in early diagnosis of cancer patients. Int J Clin Exp Med, 2015. 8(9): p. 16890-8.
[58]. Zong, W., et al., Evaluating the diagnostic and prognostic value of serum long non-coding RNA CTC-497E21.4 in gastric cancer. Clinical Chemistry and Laboratory Medicine (CCLM), 2019. 57(7): p. 1063-1072.
[59]. Anderson, B.W., et al., Detection of Gastric Cancer with Novel Methylated DNA Markers: Discovery, Tissue Validation, and Pilot Testing in Plasma. Clinical Cancer Research, 2018. 24(22): p. 5724-5734.
[60]. Balgkouranidou, I., et al., Assessment of SOX17 DNA methylation in cell free DNA from patients with operable gastric cancer. Association with prognostic variables and survival. Clinical Chemistry and Laboratory Medicine, 2013. 51(7).
[61]. Balgkouranidou, I., et al., Prognostic role of APC and RASSF1A promoter methylation status in cell free circulating DNA of operable gastric cancer patients. Mutation Research/Fundamental and Molecular Mechanisms of Mutagenesis, 2015. 778: p. 46-51.
[62]. Cao, C., L. Chang and Q. Wu, Circulating methylated Septin 9 and ring finger protein 180 for noninvasive diagnosis of early gastric cancer. Translational Cancer Research, 2020. 9(11): p. 7012-7021.
[63]. Chen, L., et al., Hypermethylated FAM5C and MYLK in serum as diagnosis and pre-warning markers for gastric cancer. Dis Markers, 2012. 32(3): p. 195-202.
[64]. Han, J., et al., Circulating Methylated MINT2 Promoter DNA Is a Potential Poor Prognostic Factor in Gastric Cancer. Digestive Diseases and Sciences, 2014. 59(6): p. 1160-1168.

[65]. Hideura, E., et al., Blood Free-Circulating DNA Testing of Methylated RUNX3 Is Useful for Diagnosing Early Gastric Cancer. Cancers, 2020. 12(4): p. 789.

[66]. Lee, H.S., et al., Circulating Methylated Septin 9 Nucleic Acid in the Plasma of Patients with Gastrointestinal Cancer in the Stomach and Colon. Translational Oncology, 2013. 6(3): p. 290-IN4.

[67]. Li, H., et al., Feasibility and reproducibility of a plasma-based multiplex DNA methylation assay for early detection of gastric cancer. Pathology - Research and Practice, 2022. 238: p. 154086.

[68]. Li, W., et al., Detection of OSR2, VAV3, and PPFIA3 Methylation in the Serum of Patients with Gastric Cancer. Disease Markers, 2016. 2016: p. 1-7.

[69]. Lin, Z., et al., Combined Detection of Plasma ZIC1, HOXD10 and RUNX3 Methylation is a Promising Strategy for Early Detection of Gastric Cancer and Precancerous Lesions. Journal of Cancer, 2017. 8(6): p. 1038-1044.

[70]. Ling, Z.Q., et al., Circulating Methylated XAF1 DNA Indicates Poor Prognosis for Gastric Cancer. PLoS One, 2013. 8(6): p. e67195.

[71]. Miao, J., et al., Feasibility of Plasma-Methylated SFRP2 for Early Detection of Gastric Cancer. Cancer Control, 2020. 27(2): p. 107327482092255.

[72]. Pimson, C., et al., Aberrant methylation of PCDH10 and RASSF1A genes in blood samples for non-invasive diagnosis and prognostic assessment of gastric cancer. PeerJ, 2016. 4: p. e2112.

[73]. Saliminejad, K., et al., Methylation Analysis of P16, RASSF1A, RPRM, and RUNX3 in Circulating Cell-Free DNA for Detection of Gastric Cancer: A Validation Study. Avicenna J Med Biotechnol, 2020. 12(2): p. 99-106.

[74]. Xu, J., et al., A combination of methylation and protein markers is capable of detecting gastric cancer detection by combined markers. Epigenomics, 2021. 13(19): p. 1557-1570.

[75]. Yang, Q., et al., Promoter hypermethylation of BCL6B gene is a potential plasma DNA biomarker for gastric cancer. Biomarkers, 2013. 18(8): p. 721-725.

[76]. Yu, J.L., et al., Methylated TIMP-3 DNA in body fluids is an independent prognostic factor for gastric cancer. Arch Pathol Lab Med, 2014. 138(11): p. 1466-73.

[77]. Zhang, H., et al., Detection of aberrant hypermethylated spastic paraplegia-20 as a potential biomarker and prognostic factor in gastric cancer. Medical Oncology, 2014. 31(2).

[78]. Zhang, X., et al., Detection of aberrant promoter methylation of RNF180, DAPK1 and SFRP2 in plasma DNA of patients with gastric cancer. Oncol Lett, 2014. 8(4): p. 1745-1750.

[79]. Zhao, L., et al., Plasma-Methylated SEPT9 for the Noninvasive Diagnosis of Gastric Cancer. J Clin Med, 2022. 11(21).
